# Clinical value of circulating tumor cells and hematological parameters in 617 Chinese patients with colorectal cancer: retrospective analysis

**DOI:** 10.1186/s12885-023-11204-7

**Published:** 2023-07-28

**Authors:** Yuhao He, Xinxin He, Yubo Zhou, Shanshan Luo

**Affiliations:** 1 Department of Comprehensive Internal Medicine, Guangxi Medical University Cancer Hospital, Nanning, China; 2 Department of Gastrointestinal Surgery, Guangxi Medical University Cancer Hospital, Nanning, China; 3 Department of Guangxi Clinical Research Center for Colorectal Cancer, Nanning, China; 4 Department of Geriatrics, Endocrinology and Metabolism, The First Affiliated Hospital of Guangxi Medical University, Nanning, China

**Keywords:** Circulating tumor cells, Colorectal cancer, Hematologic parameters

## Abstract

**Background:**

Circulating tumor cells (CTCs) have been a non-invasive technique which allows investigation of tumor characteristics. The purpose of this study was to investigate the relationship between circulating tumor cells and colorectal cancer.

**Methods:**

The clinical data of 617 patients with colorectal cancer from October 2019 to March 2022 were retrospectively collected to analyze the correlation between CTCs and clinicopathologic characteristics.

**Results:**

The CTCs value increased with the progression of Tumor(T) stage,Metastasis(M) stage and Tumor Node Metastasis(TNM) stage (*P* < 0.05), but not with Node (N) stage (*P* > 0.05). Binary logistic regression analysis showed that CTCs, CEA, CA125 and CA199 were independent risk factors for CRC metastasis. Compared with CTCs, CEA, CA125 and CA199, the Logistic model had the highest AUC (AUC = 0.778,95%CI: 0.732–0.824), and the specificity and sensitivity were 82.9% and 63.2%, respectively. After operation, chemo-radiotherapy and other treatment for CRC, CTCs and CEA were significantly decreased compared with before treatment (*P* < 0.05). In addition, Spearman Correlation showed significant correlation between CTCs and IgG (*P* = 0.000).

**Conclusion:**

CTCs, CEA, CA125 and CA199 were independent risk factors for CRC metastasis.CTCs can be used for the prediction of tumur metastasis, and the evaluation of therapeutic effect.

## Background

Colorectal cancer (CRC) is one of the most common malignant tumors in China, and its incidence ranks among the top three in China [1]. Despite great advances in the diagnosis and treatment of colorectal cancer, metastatic colorectal cancer (mCRC) remains the second most common cause of cancer-related death in the United States [2]. How to diagnose CRC at an early stage and screen patients for feasible radical surgery is a hot topic in current research. Traditional tissue biopsy is invasive, sometimes only a small number of samples can be obtained, which cannot reflect the heterogeneity of the tumor or dynamically monitor the progress of the tumor. Circulating tumor cells (CTCs) are epithelial cancer cells that have the ability to move, migrate and invade blood vessels afterepithelial-mesenchymal transition.CTCs have been a useful non-invasive liquid biopsy technique in recent years [3–6]. CTCs have several advantages, including easy sampling, effective monitoring, and dynamic evaluation of treatment. Therefore, in this study, medical records of 617 patients with colorectal cancer were retrospectively analyzed to investigate the relationship between circulating tumor cells and colorectal cancer.

## Methods

### Study design and patient cohort

Clinical data of 617 patients with colorectal cancer from the Guangxi Medical University Cancer Hospital between October 2019 and March 2022 were collected. Including age, sex, CTCs, CEA, CA125, CA199, CA724, Absolute Neutrophil Count/ Absolute Lymphoblastic Counter, IgG, IgM, IgA, C3, C4, T lymphocytes, helper T lymphocytes, inhibitory T lymphocytes, natural killer cells, B lymphocytes, tumor primary site, degree of differentiation, The depth of invasion, lymph node metastasis, distant organ metastasis, clinical staging and other data.

### Inclusion and exclusion criteria

Inclusion criteria were as follows: (1) Complete clinical data; (2) It was confirmed by histopathology as colorectal cancer. (3)Patients signed the informed consent form.Exclusion criteria were as follows: (1) Combined history of other malignant tumors;(2) Severe cardiovascular disease.

### Detection of CTCs

**Fig. 1:**
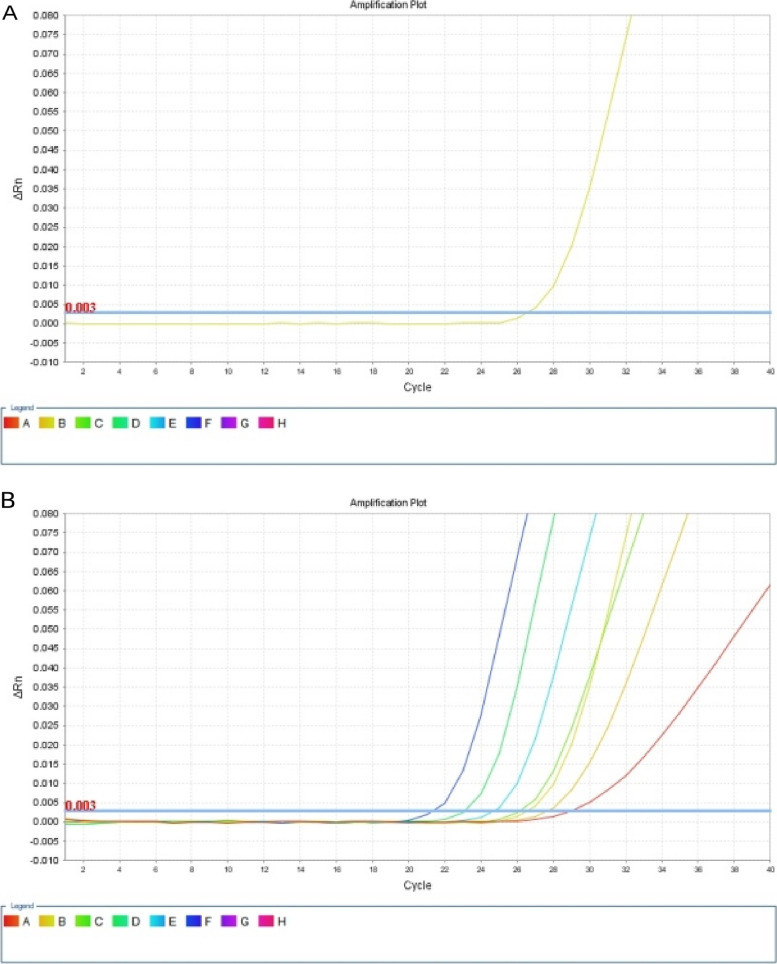
Representative images of the detection of CTCs in the peripheral blood of patients with colorectal cancer. **A, B** demonstrates the quantitative PCR analysis

CTCs detection time point: The first CTCs detection was performed before the beginning of surgery. The second examination was performed at the 12th week post-operatively(median time point). A CytoploRare^®^Circulating CRC cell kit was provided byGenoSaber Biotech Co., Ltd. (Shanghai, China). The kit consisted of three components: CTCs immu-beads enrichment ,targeted probe labeling and fluorescence quantitative PCR. The CTCs immunomagnetic beads enrichment component included red cell lysis buffer,incubation buffer, anti-CD45 leukocyte depletion magnetic beads, washing buffer, labeling buffer, stripping buffer, and neutralization buffer. The detection and quantification component included PCR reaction buffer, primers, deionized water, positive and negative cell controls, PCR controls, and standards. The primer sequences were listed as follows: RT primer, 59-CTCAACTGGTGTCGTGGAGTCGGCAATTCAGTTGAGGGTTCTAA-39; Forward primer, 59-TATGATTATGAGGCATGA-39; Reverse primer, 59-GGTGTCGTGGAGTCG-39; Taqman Probe, 59-FAM-CAGTTGAGGGTTC-MGB-39.Following the manufacturer’s instructions, CTCs were enriched by dissolving red blood cells and depleting white blood cells in a 3ml blood sample with immunomagnetism. Enriched CTCs were labeled with tumor-specific ligands, folic acid, and synthetic oligonucleotide conjugates. After labeling, the enriched CTCs were cleaned thoroughly to remove unbound conjugants. Subsequently, the binding was specifically peeled from the surface of the CTC and collected for quantitative PCR analysis. Before amplification, conjugates are first annealed and extended on RT primers. The Taqman probe was then used for amplification and quantitative PCR analysis. In the method described above, CTCs were identified as FR-positive by folic acid ligated oligonucleotide labeling (Fig. 1).

### Statistical analysis

The data of CTCs and tumor markers did not correlate with normal distribution, and were expressed in the form of median and quartile interval. The Mann-Whitney U test was used to compare CTCs data between two groups, and the Kruskal-Wallis test was used to compare CTCs data between multiple groups. Wilcoxon rank sum test was used to compare the groups before and after treatment. Binary Logistic regression analysis was used to screen the risk factors of distant CRC metastasis and establish Logistic prediction model .Receiver operating characteristic curve was plotted and the diagnostic efficiency of each curve was compared to determine the optimal diagnostic value. All statistical analyses were performed using SPSS 24.0 (IBM Corp., Armonk, NY, USA), with a two-sided *P* < 0.05 considered statistically significant.

## Results

CTCs increased with the deepening of infiltration depth, distant organ metastasis, and progression of TNM stage, with statistical significance (*P* < 0.05), but no statistical significance was found in age, gender, tumor site, degree of differentiation, and lymph node metastasis (*P* > 0.05), as shown in Table 1.

**Table 1:** Analysis of clinicopathologic characteristic parameters of colorectal cancer

Parameter	classification	N	CTCs(FU/mL)			*P*
			25%th	Median	75%th	
Age	≥60	325	9.50	10.70	14.25	0.216
	≥ 60	292	9.70	11.70	15.07	
Gender	Male	378	9.50	10.90	14.50	0.104
	Female	239	9.70	11.70	15.10	
Location	Right Hemicolon	141	9.85	11.50	14.70	0.151
	Left Hemicolon	166	9.65	11.70	15.75	
	Rectum	310	9.50	10.70	14.10	
Differentiation	Poor	32	8.33	10.50	15.68	0.709
	Moderate and well	585	9.60	11.20	14.70	
T stage	T1-T2	116	8.30	10.20	13.28	0.003
	T3-T4	501	9.80	11.40	14.90	
N stage	N0	289	9.50	10.70	14.25	0.220
	N1 + N2	328	9.70	11.70	15.08	
M stage	M0	462	9.30	10.70	13.70	0.000
	M1	155	10.20	13.10	16.50	
TNM stage	I-II	240	9.30	10.50	13.20	0.000
	III-IV	377	9.75	11.90	15.50	

*Abbreviations*: *CTCs* Circulating tumor cells, *TNM* Tumor-Node-Metastasis

CTCs ≥ 11.85FU/3ml (OR = 1.090, 95%CI: 1.029 ~ 1.155), CEA ≥ 4.87ng/ml (OR = 1.007, 95%CI: 1.003 ~ 1.010), CA125 ≥ 19.90U/ml (OR = 1.004, 95%CI: 1.000 ~ 1.008), CA199 ≥ 26.55U/ml (OR = 1.002, 95%CI: 1.001 ~ 1.002) were independent influencing factors of distant CRC metastasis (all *P* < 0.05), as shown in Table 2.

**Table 2:** Logistic regression analysis of CRC distant metastasis

	Univariate analysis		Multivariate analysis	
	*OR*(95%*CI*)	*P*	*OR*(95%*CI*)	*P*
CTCs/(FU/3ml)				
< 11.85	1.0		1.0	
≥ 11.85	1.137(1.081 ~ 1.196)	0.000	1.090(1.029 ~ 1.155)	0.003
CEA/(ng/ml)				
< 4.87	1.0		1.0	
≥ 4.87	1.010(1.006 ~ 1.013)	0.000	1.007(1.003 ~ 1.010)	0.000
CA125/(U/ml)				
< 19.90	1.0		1.0	
≥ 19.90	1.008(1.004 ~ 1.013)	0.000	1.004(1.000 ~ 1.008)	0.033
CA199/(U/ml)				
< 26.55	1.0		1.0	
≥ 26.55	1.003(1.002 ~ 1.003)	0.000	1.002(1.001 ~ 1.002)	0.000

*Abbreviations*: *CTCs* Circulating tumor cells, *CEA* Carcinoembryonic antigen, *CA125* Carbohydrate antigen 125, *CA199* Carbohydrate antigen 199

**Fig. 2:**
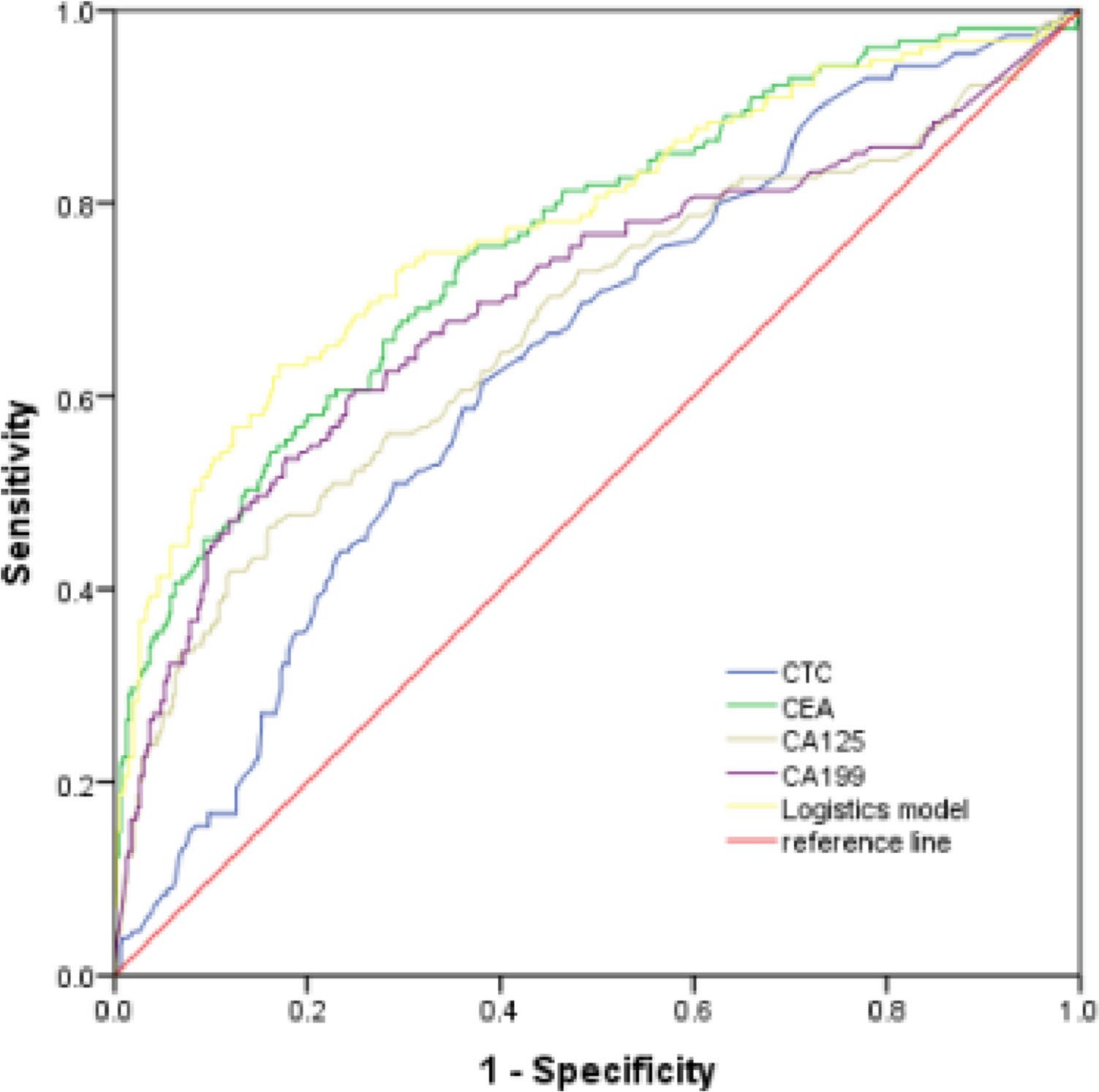
The ROC curves of CTCs, CEA, CA125,CA199 and Logistic model

The best cutoff value of CTCs in the diagnosis of CRC distant metastasis was 11.85FU/3ml, with a specificity of 61.9% and a sensitivity of 61.3%. The optimal cut-off value, specificity and sensitivity of CEA were 4.87ng/ml, the specificity was 64.3% and the sensitivity was74.2%. The best truncation value of CA125 was 19.90ng/mL, the specificity was 84.0% and the sensitivity was 46.5%. The best truncation value of CA199 was 26.55ng/mL, the specificity was 82.3%, and the sensitivity was 53.5%, as shown in Table 3. CEA (AUC = 0.761) > CA199 (AUC = 0.703) > CA125 (AUC = 0.674) > CTCs (AUC = 0.639). CTCs, CEA, CA125 and CA199 are divided into high and low groups according to the best truncation value to construct the Logistics regression model for predicting CRC distant metastasis. Logistic model=-2.767 + 0.086*CTCs + 0.007*CEA + 0.004*CA125 + 0.002*CA199. Logistic regression model had the highest AUC (AUC = 0.778,95%CI: 0.732–0.824), and the specificity and sensitivity were 82.9% and 63.2%, respectively, as shown in Table 3; Fig. 2.

**Table 3:** Diagnostic effectiveness of each factor of CRC distant metastasis

	AUC	cutoff value value	sensitivity	specificity	95%*CI*	*P*
CTCs/(FU/3ml)	0.639	11.85	61.3	61.9	0.590 ~ 0.688	0.000
CEA/(ng/ml)	0.761	4.87	74.2	64.3	0.716 ~ 0.807	0.000
CA125/(U/ml)	0.674	19.90	46.5	84.0	0.620 ~ 0.727	0.000
CA199/(U/ml)	0.703	26.55	53.5	82.3	0.650 ~ 0.756	0.000
Logistic model	0.778		63.2	82.9	0.732 ~ 0.824	0.000

*Abbreviations*: *CTCs* Circulating tumor cells, *CEA* Carcinoembryonic antigen, *CA125* Carbohydrate antigen 125, CA199 Carbohydrate antigen 199

Among the 617 CRC patients, 145 were reviewed for CTCs, CEA, CA125, CA199 and CA724 after treatment (including 116 cases of surgery and 29 cases of radiotherapy and chemotherapy). The median values of CTCs and CEA after treatment were significantly decreased compared with those before treatment (*P* < 0.05). There was no significant difference in CA125, CA199 and CA724 before and after CRC treatment (*P* > 0.05), as shown in Table 4

**Table 4:** Comparison of indexes of 145 CRC patients before and after treatment

	Before	After	*P*
CTCs/(FU/3ml)	11.70	9.30	0.000
CEA/(ng/ml)	5.45	2.92	0.000
CA125/(U/ml)	12.00	13.80	0.060
CA199/(U/ml)	16.10	10.10	0.061
CA724/(U/ml)	1.95	2.00	0.959

*Abbreviations*: *CTCs* Circulating tumor cells, *CEA* Carcinoembryonic antigen, *CA125* Carbohydrate antigen 125, *CA199* Carbohydrate antigen 199, *CA724* Carbohydrate antigen 724

CTCs and IgG were significantly correlated (*P* < 0.05). There was no significant correlation between CTCs and Absolute Neutrophil Count/ Absolute Lymphoblastic Counter, IgM, IgA, C3, C4, T lymphocytes, helper T lymphocytes, inhibitory T lymphocytes, natural killer cells and B lymphocytes (*P* > 0.05). as shown in Table 5.

**Table 5:** Relationship between CTCs and immune indexes

immune indexes	Correlation coefficient	P
IgG	0.142^*^	0.000
ANC/LYMF	0.068	0.093
IgM	-0.009	0.819
IgA	0.028	0.497
C3	0.064	0.112
C4	0.024	0.555
T lymphocytes	-0.061	0.130
helper T lymphocyte	-0.070	0.084
inhibitory T lymphocytes	-0.035	0.392
natural killer cells	0.035	0.391
B lymphocytes	0.015	0.706

*Abbreviations*: *IgG* Immunoglobulin G, *ANC/LYMF* Absolute Neutrophil Count/ Absolute Lymphoblastic Counte, *IgM* Immunoglobulin M, *IgA* Immunoglobulin A, *C3* Complement 3, *C4* Complement 4

*p<0.01

## Discussion

With the progress of research, circulating tumor cell detection has become a relatively useful non-invasive liquid biopsy technique in recent years, and detection and counting of CTCs can well predict the malignancy degree and metastasis risk of colorectal cancer, which has important diagnostic and the evaluation of therapeutic effect value [7–9]. In this study, CTCs value increased with the deepening of tumor invasion depth, distant organ metastasis, and the progression of TNM staging (*P* < 0.05), but was not correlated with lymph node metastasis (*P* > 0.05). This is consistent with the study of Zhong et al. [10], which showed that CTCs can well predict the depth of colorectal cancer invasion, the possibility of metastasis formation in the distal organ, and tumor staging.

CRC morbidity and mortality are increasing year by year. Despite some advances in molecular targeted therapy and immunotherapy for CRC in recent years, radical surgery is still the preferred treatment for resectable colorectal cancer patients. How to diagnose CRC early and screen out patients with feasible radical operation is a hot topic in current research. Previous studies have shown that tumor markers CEA, CA125 and CA199 are closely related to the diagnosis and prognosis assessment of CRC [11, 12], and the staging of CRC is closely related to the value of CTCs [3, 8]. This study combined the four factors of CTCs, CEA, CA125 and CA199. A Logistic model for the diagnosis of CRC distant metastasis was established. The AUC of Logistic model was 0.778, and the sensitivity and specificity were 63.2% and 82.9%, respectively. This indicates that the Logistic model is of high value in the diagnosis of CRC distant metastasis.

CEA is a prognostic tumor marker for CRC recommended by current guidelines, which is used for tumor diagnosis, evaluation of therapeutic effect and tumor recurrence. The CTCs detection method has high sensitivity and specificity, and can be applied to the evaluation of therapeutic effect. If the number of CTCs increases or remains unchanged, the treatment is considered ineffective, while if the number of CTCs decreases, the treatment may be effective [3]. In this study, after the effective treatment of CRC, such as surgery and chemoradiotherapy, both CTCs and CEA decreased significantly compared with that before treatment (*P* < 0.05), indicating that CTCs detection can well evaluate the therapeutic effect of surgery, chemoradiotherapy and other treatments. The advantages of CTCs testing are that it is non-invasive and easy to sample, and patient compliance is relatively good. We can conduct multiple tests during treatment to adjust the treatment regimen in real time according to its changes.

In the current literature, few studies have evaluated the relationship between immunological parameters in blood tests and CTCs. The process of CTCs formation and metastasis mainly involves several steps [13]: tumor cell release, immune escape, adhesion to blood vessels and exudation from blood vessels to form distant metastasis. Upon reaching an appropriate niche, CTCs undergo mesenchymal-epithelial transition (MET), reacquire stem cell properties and form a new metastatic site. The interaction of CTCs with the immune system plays an important role in these processes.In this study, there was a significant correlation between CTCs and IgG (*P* < 0.05), which is a new finding different from previous studies. Human immunoglobulin G(IgG) is the main component of human serum antibodies, accounting for about 75% of the immunoglobulin and 10–20% of the total plasma protein. Studies have shown that high IgG antibody level is correlated with the promotion of tumor cell proliferation and invasion and poor clinical prognosis of corresponding tumor patients [14–16]. Niu et al. [17] showed that IgG synthesized by colorectal cancer cells is involved in the occurrence and growth of colorectal cancer. Our study also had some limitations. As a retrospective study, due to the short time span of the study, postoperative follow-up information was not collected and the survival analysis of patients with colorectal cancer was not assessed based on CTCs values. May undermine the clinical significance of the study. However, we believe that the results of this study are accurate, because of the large sample size of the study, our study is still representative.

## Conclusion

CTCs, CEA, CA125 and CA199 were independent risk factors for CRC metastasis.CTCs can be used for the prediction of tumur metastasis, and the evaluation of therapeutic effect.

## Data Availability

The datasets used and/or analysed during the current study are available from the corresponding author upon reasonable request.

## Abbreviations

CTCs: Circulating tumor cells
TNM: Tumor-Node-Metastasis
CEA: Carcinoembryonic antigen
CA125: Carbohydrate antigen 125
CA199: Carbohydrate antigen 199
CA724: Carbohydrate antigen 724
IgG: Immunoglobulin G
ANC/LYMF: Absolute Neutrophil Count/Absolute Lymphoblastic Counter
IgM: Immunoglobulin M
IgA: Immunoglobulin A
C3: Complement 3
C4: Complement 4
